# Phase Lag Analyses on Ictal Scalp Electroencephalography May Predict Outcomes of Corpus Callosotomy for Epileptic Spasms

**DOI:** 10.3389/fneur.2020.576087

**Published:** 2020-12-15

**Authors:** Masayoshi Oguri, Tohru Okanishi, Sotaro Kanai, Shimpei Baba, Mitsuyo Nishimura, Kaoru Ogo, Takashi Himoto, Kazuo Okanari, Yoshihiro Maegaki, Hideo Enoki, Ayataka Fujimoto

**Affiliations:** ^1^Department of Medical Technology, Kagawa Prefectural University of Health Sciences, Takamatsu, Japan; ^2^Division of Child Neurology, Faculty of Medicine, Institute of Neurological Sciences, Tottori University, Yonago, Japan; ^3^Department of Child Neurology, Seirei-Hamamatsu General Hospital, Hamamatsu, Japan; ^4^Department of Clinical Laboratory, University of Tsukuba Hospital, Tsukuba, Japan; ^5^Department of Pediatrics, Faculty of Medicine, Oita University, Yufu, Japan; ^6^Comprehensive Epilepsy Center, Seirei-Hamamatsu General Hospital, Hamamatsu, Japan

**Keywords:** corpus callosotomy, cross-frequency analysis, electroencephaloagraphy (EEG), epileptic spasms (ES), phase lag analysis, computer analysis, pre-surgical evaluation of epilepsy

## Abstract

**Objective:** We aimed to clarify the patterns of ictal power and phase lag among bilateral hemispheres on scalp electroencephalography (EEG) recorded pre-operatively during epileptic spasms (ESs) and the correlation with the outcomes following corpus callosotomy.

**Methods:** We enrolled 17 patients who underwent corpus callosotomy for ESs before 20 years of age. After corpus callosotomy, seven patients did not experience further ESs (favorable outcome group), and the remaining 10 patients had ongoing ESs (unfavorable outcome group). We used pre-operative scalp EEG data from monopolar montages using the average reference. The relative power spectrum (PS), ictal power laterality (IPL) among the hemispheres, and phase lag, calculated by the cross-power spectrum (CPS) among symmetrical electrodes (i.e., F3 and F4), were analyzed in the EEG data of ESs from 143 pre-operative scalp video-EEG records. Analyses were conducted separately in each frequency band from the delta, theta, alpha, beta, and gamma range. We compared the means of those data in each patient between favorable and unfavorable outcome groups.

**Results:** Among all frequency bands, no significant differences were seen in the individual mean relative PSs in the favorable and unfavorable outcome group. Although the mean IPLs in each patient tended to be high in the unfavorable outcome group, no significant differences were found. The mean CPSs in the delta, theta, and gamma frequency bands were significantly higher in the unfavorable than in the favorable outcome group. Using the Youden index, the optimal cutoff points of those mean CPS values for unfavorable outcomes were 64.00 in the delta band (sensitivity: 100%, specificity: 80%), 74.20 in the theta band (100, 80%), and 82.05 in the gamma band (100, 80%). Subanalyses indicated that those CPS differences originated from pairs of symmetrical electrodes in the bilateral frontal and temporal areas.

**Significance:** Ictal power and laterality of the ictal power in each frequency band were not associated with the outcomes of CC; however, the phase lags seen in the delta, theta, and gamma frequency bands were larger in the unfavorable than in the favorable outcome group. The phase lags may predict outcomes of CC for ESs on pre-surgical scalp-ictal EEGs.

## Introduction

Epileptic spasms (ESs) are seizures leading to muscular contraction, typically involving the axial muscles and proximal limb segments (1); they appear mainly in patients with West syndrome. The patterns of electroencephalography (EEG) and electromyography (EMG) activity observed in these spasms are similar (2). Ictal EEG findings in these patients comprise three contiguous phases: (1) 15- to 20-Hz spindle-like fast activity in posterior areas, (2) diffuse polyphasic delta/theta waves, and (3) electrodecremental activity (1, 3). Diffuse polyphasic delta/theta waves occur in 100%, and electrodecremental activity occurs in 70% of patients with ESs (4). Ictal EMG findings in these patients have rhombus or diamond shapes. When electroencephalographic/video monitoring first came into clinical use, several studies investigated ictal EEG patterns of ESs, including isolated spindle-like activity, high-amplitude slow wave, the spindle-like activity followed by the slow wave, and decremental activity, which follows the slow wave (1). In recent studies, computer-based frequency analysis has been adapted to estimate the ictal-scalp EEG of ESs. The scalp EEG data of ESs showed components of wide frequencies from delta to high gamma bands, and the coupling of high gamma and slow wave EEG components associated with the response to medical treatment (5).

Corpus callosotomy (CC) is a valuable palliative surgical option for patients with generalized seizures with diffuse or multifocal epileptic discharges (6, 7). Some reports have indicated that CC exerts beneficial effects in patients with ESs (8–11). Taking the results of these studies collectively, ESs were eliminated after CC in 42 of 87 patients. Previous studies have attempted to elucidate the prognostic factors following CC for ESs, finding good prognostic factors to include the absence of imaging abnormalities, normal development at the time of surgery, no background etiology, and performance of total callosotomy (11–15). A developmental delay before the onset of epilepsy has been shown to be associated with the worst outcomes following CC for ESs in West syndrome (9). Although electrophysiological factors that predict the outcome of CC for ESs were previously unknown, we recently found symmetrical ictal slow waves during the emergence of ESs to be associated with good outcomes following CC (16). In this study, three asymmetrical indices were identified: interhemispheric delay of negative peaks, interhemispheric ratio of amplitude for the highest positive peak, and interhemispheric ratio of slow wave duration. However, the study did not include the analyses for ictal waves faster than alpha rhythms, and computer-based frequency analyses are necessary to identify more objective and detailed associations between interhemispheric brain activity and the effectiveness of CC.

The aim of the current ictal EEG study was to use computer-based quantitative analysis to clarify the power of ictal period, laterality of ictal power, and phase lag among bilateral hemispheres in the variable frequency bands.

## Materials and Methods

### Patients

The studies involving human participants were reviewed and approved by the Seirei Hamamatsu General Hospital and Tottori University Hospital Clinical Research Review Committees. We retrospectively collected patients' clinical data from medical charts and reviewed the video-EEG recordings. Written informed consent to participate in this study was provided by the participants' legal guardian/next of kin.

Seventeen patients (female: 2; male: 15) with epilepsy were screened from patients admitted to the Seirei-Hamamatsu General Hospital between 2010 and 2017. The inclusion criteria for this study were the same as those in our previous study (16), which were as follows: (1) patients undergoing CC between January 2008 and December 2017 at the Seirei-Hamamatsu General Hospital, (2) CC performed before the age of 20 years, (3) the patient's main seizure type was ES, (4) the patient received ictal EEG recordings prior to CC, and (5) a follow-up period following CC of more than 6 months. We defined ES as a seizure (1) leading to contractions in axial muscles, (2) presenting with ictal EEGs containing polyphasic high-voltage delta/theta waves, and (3) presenting with an ictal electromyogram showing rhombus or diamond shapes. We excluded patients with inappropriate EEG recordings, such as those with misplaced EEG electrodes or serious surgical complications.

### Clinical Profiles

We reviewed the patients' clinical profiles, including the sex, age at epilepsy onset, seizure types prior to CC, classification of epilepsy or epilepsy syndrome prior to CC, total number of antiepileptic drugs (AEDs) prescribed before CC, frequency of ESs, etiology, age at CC, procedures for CC, and follow-up period.

The seizure outcomes of ESs after CC were assessed based on the Engel's classification at the last follow-up. We classified the patients into a favorable outcome group (seizure free = Engel classification I) and an unfavorable outcome (residual seizures = Engel classifications II to IV).

### Scalp Video-EEG Recordings

Scalp video-EEGs were performed using NicoletOne or BMSI6000 (Natus Medical Incorporated, WI) for patients 1–4, 6–9, and 11–16, and Neurofax (Nihon-Kohden, Japan) for patients 5, 10, and 17. EEG was sampled at 256 Hz (patients 3, 8, 12, and 16), 400 Hz (patients 1, 2, 4, 7, 11, 14, and 15), 500 Hz (patients 5, 10, and 17), 512 Hz (patients 6 and 13), and 1,024 Hz (patient 9). Electrodes were placed according to the international 10/20 system, using at least 16 EEG channels (Fp1, Fp2, F3, F4, C3, Cz, C4, P3, P4, O1, O2, F7, F8, T3, T4, T5, and T6). The ground electrode was set attached to the frontal pole (Fpz). Electromyogram (EMG) electrodes were placed on both deltoid muscles.

### Quantitative EEG Analysis

#### Selection of Ictal EEG, Time Window, and Electrodes for the Analyses

We visually reviewed the video-EEG records, and selected ES based on the ictal EEG change of polyphasic delta or theta waves with EMG activities of rhombus or diamond shapes, coincidently occurring with clinical muscular contraction in the neck, shoulder, and/or body trunk on video recording.

Initially, the ictal record was identified visually by each muscular contraction on video and EMG recording. Then, we reviewed the ictal EEG records of polyphasic delta or theta waves, which coincided with the muscular contractions. We excluded the records that detected physiological (muscle, movement, cardiac, tongue movement, and eye movement) or non-physiological (line noise, electrode artifacts, and other equipment) artifacts.

**Figure 1:**
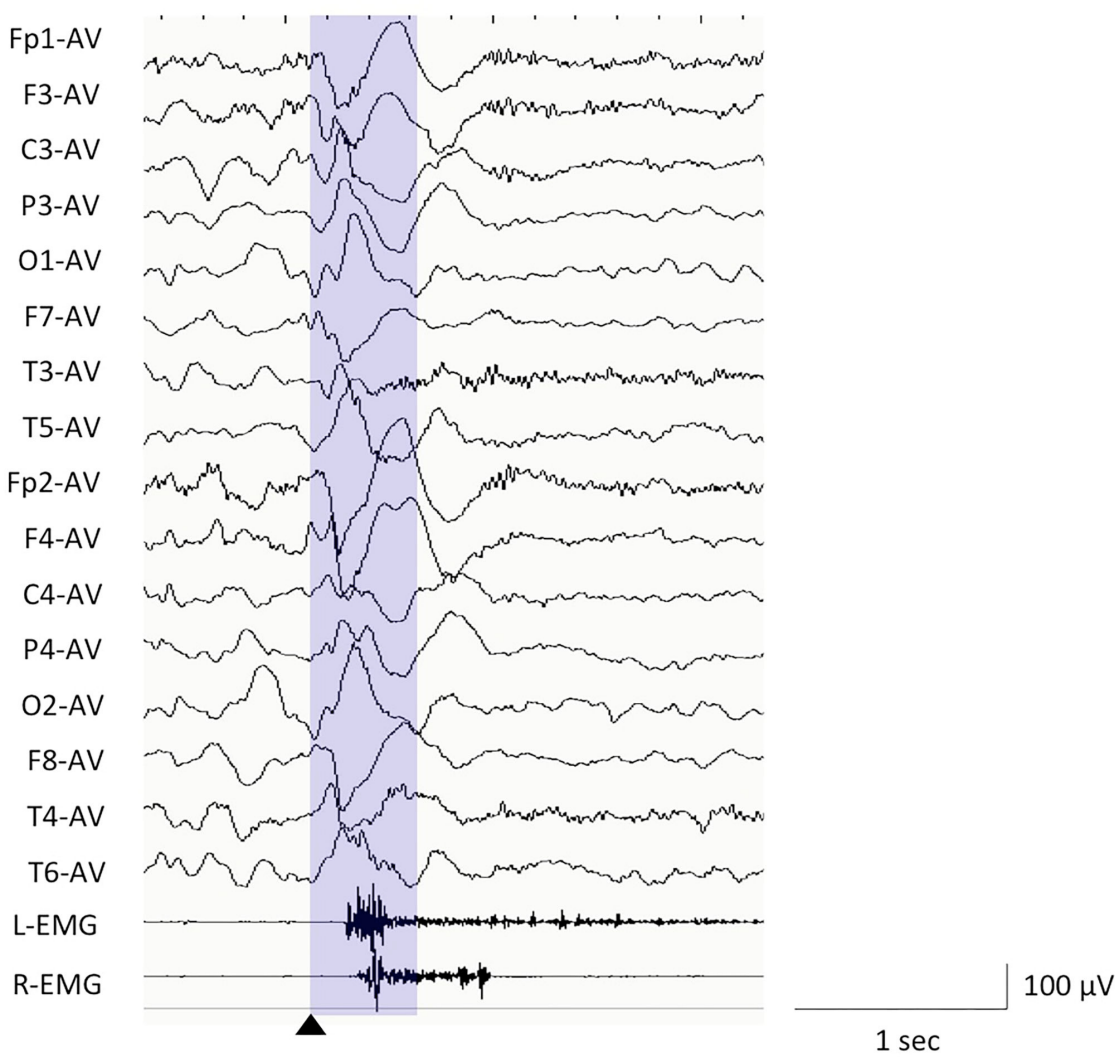
Electroencephalography (EEG) example of epileptic spasms in monopolar montages of average reference and EEG analysis window. We used the EEG data between the trigger point (0 s, black triangle) and 500 ms (purple window) for the computer analyses.

We used the EEG data of ictal polyphasic slow waves, using the average reference (Figure 1). The EEG data, which were mainly preceding, coinciding with or preceding ictal muscle contractions, and presented the typical negative–positive–negative waveform were visually selected from each record. For the visual review of EEG, the sensitivity was set at 10 μV/mm with low cut and high filter of 0.5 and 70 Hz for NicoletOne or BMSI6000 and 1.6 and 60 Hz for Neurofax, respectively. We set the trigger point (0 s) for the window of EEG analysis as the start of the negative peak. Focal spasms were defined as the ES with apparently asymmetrical or asynchronous among the movements of bilateral extremities on the video, or twice or more different amplitudes of bilateral EMG.

For the computer-based analyses, we used the EEG data with monopolar montages (Fp1, Fp2, F3, F4, C3, C4, P3, P4, O1, O2, F7, F8, T3, T4, T5, and T6), using the average reference. The EEG data were calculated using MATLAB plug-in for EEGLAB. The frequency bands were classified into delta (0.5–3.9 Hz), theta (4.0–7.9 Hz), alpha (8.0–12.9 Hz), beta (13.0–29.9 Hz), and gamma (30.0–79.9 Hz) bands.

#### Analysis for Relative Power Spectrums

We initially analyzed the powers of each ictal EEG by individual frequency band. For the quantitative analysis, the relative power spectrum (PS) was calculated using the Welch's method with a Hamming window, for frequencies between 0.5 and 79.9 Hz. We used the EEG data with monopolar montages (Fp1, Fp2, F3, F4, C3, C4, P3, P4, O1, O2, F7, F8, T3, T4, T5, and T6), using the average as reference. The PS was calculated using MATLAB plug-in for EEGLAB. First, we analyzed and averaged PS from −0 and 500 ms on each electrode in each frequency band. The frequency bands were then classified into delta (0.5–3.9 Hz), theta (4.0–7.9 Hz), alpha (8.0–12.9 Hz), beta (13.0–29.9 Hz), and gamma (30.0–79.9 Hz) bands.

We first calculated the relative PS on each electrode during an ictal EEG. Second, the relative PSs among all electrodes were averaged for each ictal EEG (mean relative PS *per seizure*). Finally, we calculated the mean of “the mean relative PSs *per seizure*” for all ictal EEGs per patient (mean relative PS in each patient). In these analyses, we separately calculated the values by each frequency band.

#### Analysis for Laterality of the Ictal Relative PS (Ictal Power Laterality)

We intended to identify the differences in the EEG PS among each pair of symmetrical electrodes. We defined “ictal power laterality (IPL)” of each ictal EEG in this study using the following formula:

$$\begin{array}{l} IPL=\frac{The\;higher\;relative\;PS\;value\;\left(on\;the\;right\;or\;left\;hemisphere\right)}{The\;lower\;relative\;PS\;value\;\left(on\;the\;other\;electrode\right)} \end{array}$$

For example, if a patient had a relative PS of 0.41 dB on Fp1 and 0.64 dB on Fp2 in the delta band during an ictal EEG, the result of IPL among the pair of Fp1 and Fp2 would be 0.64/0.41 = 1.57. We first calculated the IPL of each pair of symmetrical electrodes (Fp1 vs. Fp2, F3 vs. F4, C3 vs. C4, P3 vs. P4, O1 vs. O2, F7 vs. F8, T3 vs. T4, and T5 vs. T6) during each ictal EEG. Subsequently, we calculated the mean IPL of all pairs of symmetrical electrodes during an ictal EEG (mean IPL *per seizure*) and then calculated the mean of “the mean IPLs *per seizure*” for all ictal EEGs per patient (mean IPL in each patient). In these analyses, we separately calculated these values by each frequency band.

#### Analysis for Cross-Power Spectrum

The cross-power spectrum (CPS) estimates the degrees of the phase lag of two discrete-time signals x and y, using the Welch's averaged and modified periodogram method of spectral estimation. We used the same data selected for the PS analysis to calculate the CPS. We calculated the CPS using MATLAB software using cross-power spectrum density. The CPS is the distribution of power per unit frequency, and is defined as:

$$\begin{array}{l} Pxy\left(f\right)\;=\;\overset{⋈}{\underset{m=-⋈}{\sum }}Rxy\left(m\right)e^{-i\omega t} \end{array}$$

The cross-correlation sequence is defined as:

$$\begin{array}{l} Rxy\left(m\right)=E\left\{x_{n+m}{y*}_{n}\right\}=E\left\{x_{n}y_{n-m}\right\}, \end{array}$$

where x_n_ and y_n_ are jointly stationary random processes, –∞ < n < ∞, –∞ < n < ∞, and E {· } is the expected value operator.

The values of CPS represented ranged from 0 to 180 degrees on each frequency band. First, we calculated the absolute CPS of an ictal EEG at each pair of symmetrical electrodes (Fp1 vs. Fp2, F3 vs. F4, C3 vs. C4, P3 vs. P4, O1 vs. O2, F7 vs. F8, T3 vs. T4, and T5 vs. T6) in each ictal EEG. Second, we calculated the mean of the absolute CPS in all pairs of symmetrical electrodes in the ictal EEG (mean CPS *per seizure*). Finally, we calculated the mean of “the mean CPS *per seizure*” for all ictal EEGs per patient (mean CPS in each patient). In these analyses, we separately calculated these values by each frequency band.

We performed subanalysis of CPS to identify the electrode pairs from which the phase lags originated; we calculated the CPS at each electrode pair for each ictal EEG and calculated the mean of the CPS for each electrode pair during all ictal EEGs in each patient (mean CPS of each pair of symmetrical electrodes in each patient). During analysis, we also separately calculated them by each frequency band.

### Statistical Analyses

All data were analyzed using GraphPad Prism 6 (GraphPad Software, La Jolla, CA, USA). For comparing the data of clinical profiles, we used the Fisher's exact probability, Welch t-, and chi-square tests, as appropriate. By removing the effect of age, we used analysis of covariance for comparing the relative PS, IPL, and CPS among the outcome groups. The mean relative PS, mean IPL, and mean CPS were individually compared between the favorable and the unfavorable outcome groups using the Welch *t*-test by each frequency band.

Multiple liner regression was used to identify significant correlations between the mean CPS of each ictal EEG in each patient and the outcomes. We calculated the optimal cut-off points of CPS values in the frequency band with statistical significance for the predictive factors of unfavorable outcome using the Youden index.

A *p*-value of < 0.05 was considered to indicate statistical significance.

## Results

### Clinical Profiles

The details regarding the clinical information are shown in Table 1. The age at epilepsy onset ranged from 1 to 166 months old (mean: 23 months). The classifications of the epilepsy/epilepsy syndrome were West syndrome (*n* = 10) and combined generalized and focal epilepsy (*n* = 7). Fourteen patients developed more than two types of seizures, including tonic seizures, focal impaired awareness seizure, atonic seizures, and myoclonic seizures. The mean frequency of ES pre-operatively was 40.1 times per day (range: 5–200).

**Table 1:** Clinical profiles of patients in the favorable and unfavorable outcome groups.

	**Favorable outcome group (*n* = 7)**	**Unfavorable outcome group (*n* = 10)**	***p*-value**
Sex (boys: girls)	6:1	9:1	n.s.
Types of epilepsy syndrome			n.s.
West syndrome	2	8	
Combined generalized and focal epilepsy	5	2	
Etiology			n.s.
Structural abnormality	5	6	
Genetic/chromosomal syndrome	2	1	
Unknown	0	3	
Age at epilepsy onset [months, range (mean)]	4–166 (49)	1–13 (5)	n.s.
Total number of AEDs before CC [range (mean)]	4–8 (6.6)	6–10 (7.3)	n.s.
Frequency of ES/TS			n.s.
1–20/day	5	5	
>20/day	2	5	
Artifact free ES/TS using analysis [times, range (mean)]	5–15 (9)	4–12 (8)	n.s.
Age at CC [months, range (mean)]	45–237 (125)	17–106 (51)	0.042
Procedure of CC			n.s.
Total callosotomy	6	8	
Anterior 4/5 callostomy	1	2	
Outcomes of Engel's classification			NA
I	7	—	
II	—	2	
III	—	4	
IV	—	4	
Follow-up periods [months, range (mean)]	8–36 (17)	10–72 (26)	n.s.

AEDs, antiepileptic drugs; CC, corpus callosotomy; n.s., not significant; NA, not applicable. We used Fisher's exact probability test, Welch t-test, and chi-square test, appropriately. Tuberous sclerosis complex was classified as a structural abnormality in etiology.

The patients' etiologies were identified in 16: tuberous sclerosis complex (*n* = 5), post-acute encephalopathy (*n* = 3), post-neonatal hypoglycemia (*n* = 2), hippocampal sclerosis (n = 1), focal cortical dysplasia (*n* = 1), chemotherapy-induced leukoencephalopathy (*n* = 1), methyl-CpG binding protein 2 (*MECP2*) duplication syndrome (*n* = 1), post-neonatal hypoxic–ischemic encephalopathy (*n* = 1), and Down syndrome (*n* = 1); one patient had both TSC and acute encephalopathy, and one patient had both *MECP2* duplication syndrome and acute encephalopathy. Three patients did not have a neurologic history prior to the onset of epilepsy.

The mean age at CC was 81.4 months (range: 17–237 months). The mean follow-up period after CC was 22.1 months (range: 8–72 months). Seven patients showed a favorable outcome (Engel classification I). Ten patients showed unfavorable outcomes (Engel II in two patients, III in four patients, and IV in four patients).

Among the outcome groups, only the age at CC was significantly higher in the favorable outcome group (*p* = 0.042) than in the unfavorable outcome group. No significant differences were seen in other factors.

### Selection of Ictal EEG

In total, 143 ES were visually identified in this study. The range and the median number of ES per patient were 4–15 and 9, respectively. Fifteen EEG records of ES were excluded due to non-negligible artifacts or misplacements of EEG electrodes. For the first time, SK and MS anonymously reviewed and selected ictal EEG. They discussed and excluded 15 ES due to the artifacts or misplacements of the EEG electrodes. MO performed computer analyses for the ES data. Focal spasms were seen in seven patients. All of those patients showed unfavorable outcome.

### Relative PS

The mean relative PSs for each patient in the favorable and unfavorable outcome groups were 1.45 ± 2.07 (range: −0.60–49.56) (standard deviation: SD) and 2.52 (mean) ± 6.16 (range: −1.38–244.91) in the delta frequency band, 0.48 ± 1.71 (range: −2.80–38.79) and −0.55 ± 2.04 (range: −97.27–11.18) in the theta frequency band, −0.45 ± 0.41 (range: −6.28–1.63) and −1.97 ± 5.05 (range: −209.00–1.45) in the alpha frequency band, −0.42 ± 1.04 (range: −25.13–1.41) and 1.37 ± 4.61 (range: −8.61–192.96) in the beta frequency band, −1.00 ± 2.53 (range: −61.74–1.66) and −1.35 ± 3.99 (range: −132.61–29.65) in the gamma frequency band, respectively.

No significant differences were identified in the individual mean relative PSs in the all-frequency bands between the favorable and the unfavorable outcome groups (Table 2).

**Table 2:** The mean relative power spectrum of each patient in the favorable and unfavorable outcome groups.

**Frequency bands**	**Favorable outcome group**	**Unfavorable outcome group**	***p*-value**
Delta	1.45 ± 2.07	2.52 ± 6.16	0.277
Theta	0.48 ± 1.71	−0.55 ± 2.04	0.842
Alpha	−0.45 ± 0.41	−1.97 ± 5.05	0.107
Beta	−0.42 ± 1.04	1.37 ± 4.61	0.194
Gamma	−1.00 ± 2.53	−1.35 ± 3.99	0.531

After removing age effect, we used the Welch t-test.

### IPL

The mean IPLs in each patient in favorable and unfavorable outcome groups were 1.51 ± 0.47 (range: 0.73–2.21) and 6.37 ± 11.88 (range: 1.03–39.34) in the delta frequency band, 1.06 ± 0.55 (range: 0.06–1.70) and 1.06 ± 0.89 (range: 0.08–2.60) in the theta frequency band, 0.68 ± 0.28 (range: 0.20–1.01) and 1.14 ± 0.24 (range: 0.01–3.46) in the alpha frequency band, 1.60 ± 1.65 (range: 0.45–4.86) and 11.88 ± 30.08 (range: 0.13 97.09) in the beta frequency band, 1.21 ± 0.78 (range: 0.28–0.57) and 2.19 ± 3.50 (range: 0.30–11.74) in the gamma frequency band, respectively.

Although the mean IPLs in each patient tended to be higher in unfavorable outcome groups than in favorable groups, no significant differences were identified in the all-frequency bands among the groups (Table 3).

**Table 3:** The mean ictal power laterality using relative power results, averaging all electrodes in each patient in the favorable and unfavorable outcome groups.

**Frequency bands**	**Favorable outcome group**	**Unfavorable outcome group**	***p*-value**
Delta	1.51 ± 0.47	6.37 ± 11.88	0.430
Theta	1.06 ± 0.55	1.06 ± 0.89	0.176
Alpha	0.68 ± 0.28	1.14 ± 0.24	0.910
Beta	1.60 ± 1.65	11.88 ± 30.08	0.329
Gamma	1.21 ± 0.78	2.19 ± 3.50	0.885

After removing age effect, we used Welch t-test.

### CPS

**Figure 2:**
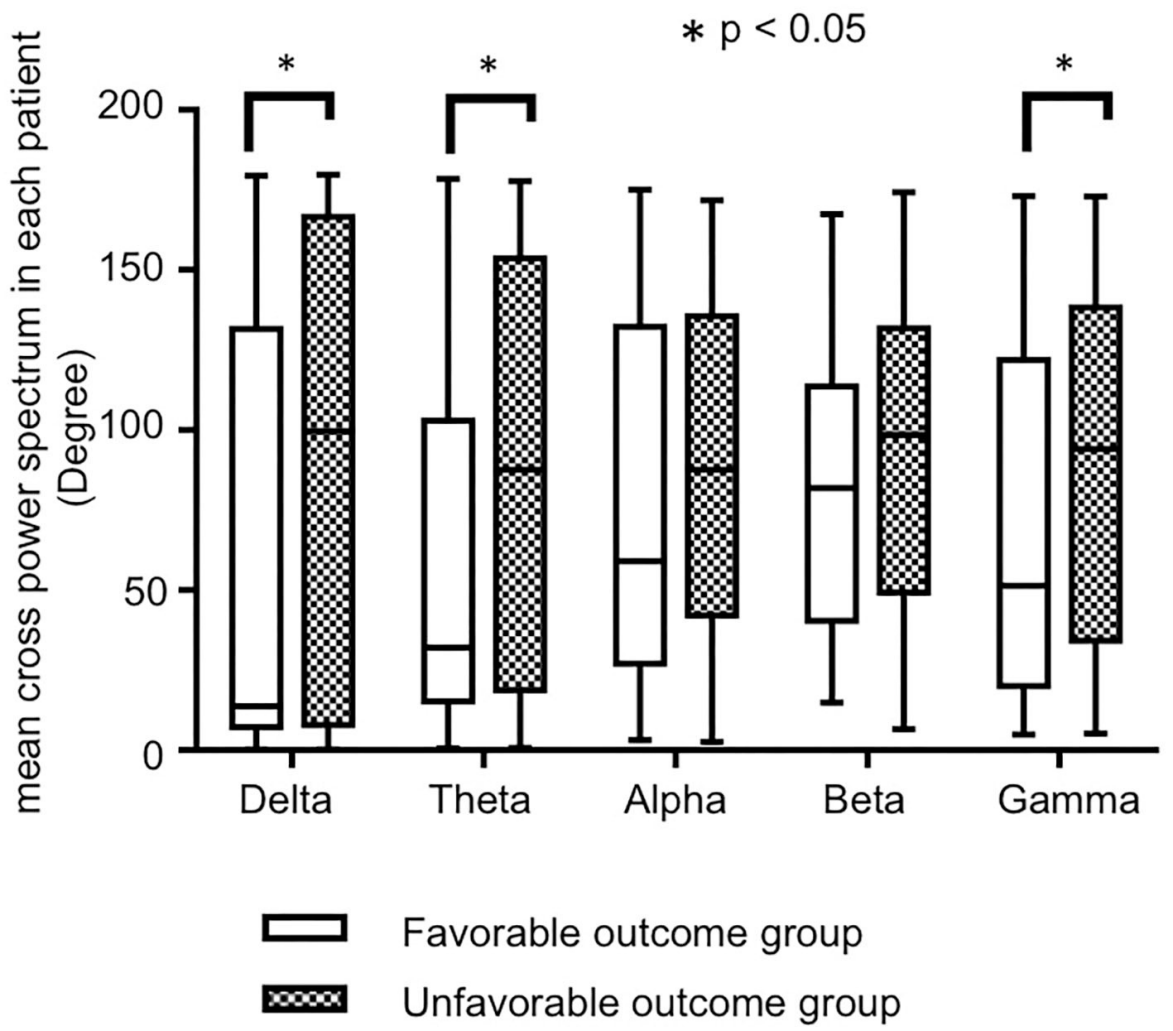
The bar charts of the mean cross-power spectrums (CPS) in each patient by each frequency band, in favorable and unfavorable outcome groups. Although the mean CPS in each patient tended to be higher in unfavorable outcome group in all frequency bands, significant differences were seen in the delta, theta, and gamma bands.

Figure 2 shows the bar charts of the mean CPS in each patient in the favorable and unfavorable outcome groups by each frequency bands The mean CPS in each patient in the favorable and unfavorable outcomes were 56.2 ± 9.2° (range: 0.32–179.5°) and 86.5 ± 8.4 (range: 0.19–179.7) degrees in the delta frequency band, 61.7 ± 7.8 (range: 0.53–178.4) and 85.0 ± 7.3 (range: 0.77–177.8) degrees in the theta frequency band, 76.7 ± 7.6 (range: 3.33–175.0) and 89.4 ± 5.7 (range: 2.6–171.7) degrees in the alpha frequency band, 79.3 ± 5.6 (range: 15.0–167.4) and 91.9 ± 5.3 (range: 6.6–174.3) degrees in the beta frequency band, 71.2 ± 7.4 (range: 5.0–173.1) and 90.9 ± 6.0 (range: 5.3–172.9) degrees in the theta frequency band, respectively. The numeric data are provided in Supplementary Table 1.

The mean CPSs in each patient in the unfavorable outcome group tended to be higher than those in the favorable outcome group in all frequency bands, and there were significant differences in the delta (*p* = 0.018), theta (*p* = 0.034), and gamma (*p* = 0.040) frequency bands.

The optimal cutoff values of the mean CPS in each patient for unfavorable outcome were 64.00 in the delta band (sensitivity: 100, specificity: 80%), 74.20 in the theta band (100, 80%), and 82.05 in the gamma band (100, 80%).

**Figure 3:**
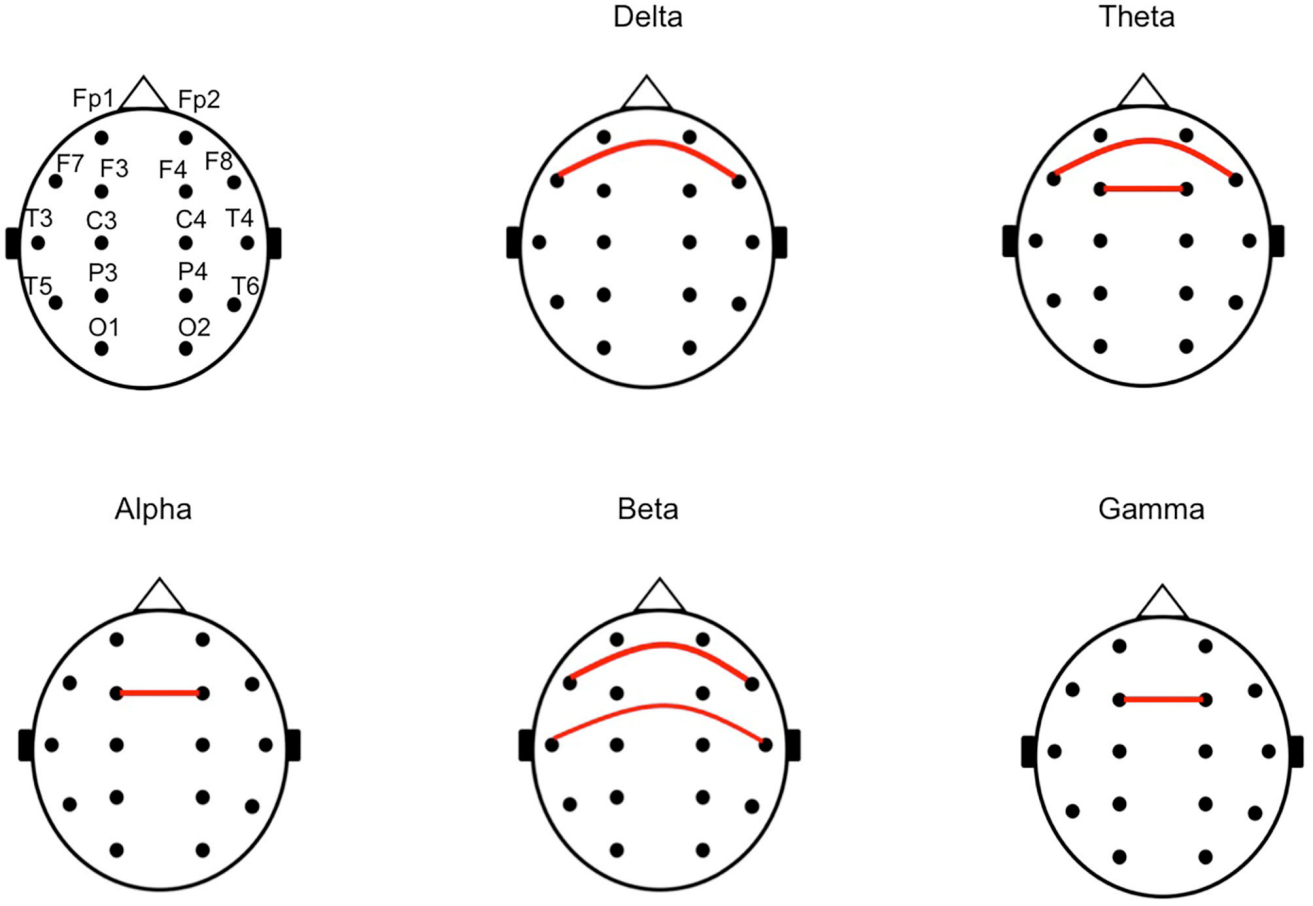
The chart of a pair of symmetrical electrodes with large phase lags in the unfavorable outcome group. The red lines indicate the pair of symmetrical electrodes with significantly large phase lags between the unfavorable and favorable outcome groups, in the analyses for mean cross-power spectrum (CPS) of each pair of symmetrical electrodes. All of the pairs with the red line showed higher CPS in the unfavorable outcome group than in the favorable outcome group.

Subsequently, we calculated the CPS of each pair of symmetrical electrodes in each patient to identify the cortical regions that generated the differences of the mean CPS in each frequency band. Overall, 72.5% (29/40) of the pairs showed higher mean CPS values in the unfavorable than in the favorable outcome group. The CPS values were significantly higher in the unfavorable outcome group than in the favorable outcome group (*p* = 0.036 at F7 vs. F8 in the delta, p = 0.041 at F3 vs. F4 and *p* = 0.041 at F7 vs. F8 in the theta, *p* = 0.048 at F3 vs. F4 in the alpha, *p* = 0.012 at F7 vs. F8 and *p* = 0.010 at T3 vs. T4 in the beta, and *p* = 0.012 at F3 vs. F4 in the gamma frequency bands). Figure 3 indicate the pairs of symmetrical electrodes with significant mean CPS differences among the outcomes groups. The numeric data are described in Supplementary Table 2.

## Discussion

### Summary of the Results

In this study, we analyzed the relative PS, IPL, and CPS by each frequency band on the scalp ictal-EEG of ES before CC. We compared the results between the patients with favorable and unfavorable outcomes after CC. No significant differences were found in terms of the relative PS and IPL between favorable and unfavorable outcome groups. Conversely, our study revealed higher CPS values regarding the delta, theta, and gamma frequency bands; these indicated phase lags of the waves of EEG among pairs of symmetrical electrodes in patients with unfavorable outcomes, compared to those with favorable outcomes. The differences in CPS tended to be apparent in the frontal and temporal areas.

### Relative PS Analyses

Previous studies on the analysis of the EEG power indicated that the emergence of gamma activity during interictal scalp EEG in patients with West syndrome positively correlated with intractableness of seizures or severity of hypsarrhythmia (17, 18). Moreover, abnormal EEGs, defined as abnormal backgrounds and focal background slowing, were indicators of poor surgical outcomes in intractable epilepsy in patients with ES on inspection (19). Regarding the studies on ictal EEG of ES, Myers et al. (20) reported that less ictal slow activity was associated with better medical treatment responses in West syndrome. Using computer-based frequency analysis, Nariai et al. (21) reported that gamma and beta activities during spasms correlated with strong ictogenesis. Although we predicted that relative PS in both slow and fast activities would be higher in the unfavorable than in the favorable outcomes groups, there were no significant differences among the groups in this study. The powers of ictal activities in ES may not associate with the outcomes of CC.

### IPL Analyses

We subsequently analyzed the IPL, which indicates laterality of powers of ictal EEG among bilateral hemispheres. In our previous report, we visually analyzed the ratio of the ictal peaks of the slow waves in bilateral hemispheres. The ictal slow waves were asymmetrical in patients with unfavorable outcomes after CC than in those with favorable outcomes (16). In this study, we compared the IPL, including delta to gamma frequency bands. However, this study did not show significant differences on any frequency bands, although the IPLs tended to be higher in the unfavorable than in the favorable outcome group. In our previous study on ictal slow waves, the electrodes with the most preceding seizure activity were selected for analysis (16). This study included all temporal and parasagittal electrodes for the analyses; the differences in the laterality may have been attenuated by averaging.

### CPS Analyses

In our previous report using visual analysis, the peak of the negative slow wave during spasms showed more time differences among bilateral hemispheres in the unfavorable outcome group (16). Using computer-based analyses, we confirmed a larger phase lag in the unfavorable than in the favorable outcome group. Significant differences were observed in both slow activities of delta or theta bands and in gamma bands. The large phase lags in the slow frequency bands in the unfavorable group may reflect the negative peak delay in our previous report (16), and the small phase lags in the patients with favorable outcomes may reflect the approximation of epileptic excitations among bilateral hemispheres via transcallosal volleys (16, 22).

We additionally analyzed the mean CPS of each symmetrical pair of electrodes in each patient, to identify the origin of the phase lags. In this study, the CPS was higher among bilateral frontal and temporal regions in the unfavorable outcome group than in the favorable outcome group. The interhemispheric connection might be predominant via the anterior part of corpus callosum in the patients with favorable outcome group.

### Study Limitations

Our study has some limitations. The number of the patients was not large, and the backgrounds of the patients were different. The results with statistical significances in this study became not significant after multiple comparisons. Larger samples are necessary for more definitive conclusion. Selection bias might occur, when we removed the ictal EEG data with muscle artifact. The sampling ratios (256 or 400 Hz) were lower than those in the previous studies (5, 21), and we could not analyze for more high frequency band of ripples (>80 Hz).

## Conclusion

In conclusion, the large phase lags of delta, theta, and gamma activities among bilateral hemispheres during ES correlated with unfavorable outcomes following CC. Asynchronous activities in ES may indicate low performance of the corpus callosum in emergent seizures or severe epileptogenicity. These computed wave analyses for ictal-scalp EEG in ES may be used pre-operatively to predict outcomes of CC for ES.

## Data Availability Statement

The raw data supporting the conclusions of this article will be made available by the authors, without undue reservation.

## Ethics Statement

The studies involving human participants were reviewed and approved by the ethical committee of Seirei Hamamatsu General Hospital. Written informed consent to participate in this study was provided by the participants' legal guardian/next of kin. Written informed consent was obtained from the minor(s)' legal guardian/next of kin for the publication of any potentially identifiable images or data included in this article.

## Author Contributions

MO designed the study, managed and analyzed the EEG data, performed statistical analyses, and wrote the manuscript. TO designed the study, performed statistical analyses, and revised the manuscript. SK analyzed the EEG data. MN analyzed and acquired the EEG data. SB, KO, TH, KO, and YM critically revised the manuscript. HE and AF discussed the interpretations of the data and revised the manuscript. All authors contributed to the article and approved the submitted version.

## Conflict of Interest

The authors declare that the research was conducted in the absence of any commercial or financial relationships that could be construed as a potential conflict of interest.
